# Solution-Friction Analytical Approximation as a Robust Model Framework for Low-Salt-Rejection Reverse Osmosis

**DOI:** 10.1021/acs.estlett.5c01130

**Published:** 2026-01-15

**Authors:** Rayan Alghanayem, Weifan Liu, Rui Chen, Shihong Lin

**Affiliations:** † Department of Chemical and Biomolecular Engineering, 5718 Vanderbilt University, Nashville, Tennessee 37235-1831, United States; ‡ Department of Chemical Engineering, College of Engineering, King Saud University, P.O. Box 800, Riyadh 11421, Saudi Arabia; § Department of Civil and Environmental Engineering, Vanderbilt University, Nashville, Tennessee 37235-1831, United States

**Keywords:** brine management, membrane, low-salt-rejection reverse osmosis, transport model

## Abstract

Low-salt-rejection reverse osmosis (LSRRO) is a promising approach for concentrating hypersaline brines using moderate hydraulic pressures, but predictive modeling of LSRRO membranes remains limited by empirical frameworks that require salinity-dependent fitting parameters. Here, we adapt and validate the solution-friction analytical approximation (SF-AA) as a simple, closed-form, and physically grounded model for low-salt-rejection membranes across a wide salinity range. Using three low-salt-rejection membranes, including a highly “leaky” membrane prepared by controlled chlorination, we conducted experiments with NaCl solutions of up to 3.64 M to measure water flux, salt rejection, and salinity-dependent salt permeability. With only three intrinsic membrane parameters, the SF-AA accurately captures the dependence of salt transport on both feed concentration and permeate flux, substantially outperforming the conventional model. These results establish the SF-AA as a robust and generalizable model for LSRRO and other membrane processes with leaky membranes and high salinity.

## Graphical abstract

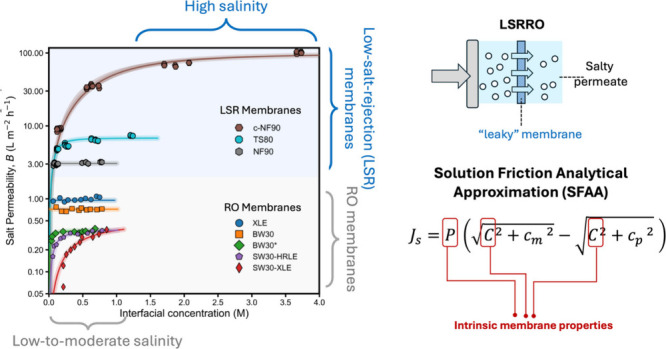

## Introduction

A large volume of hypersaline brine is generated in various industries.^1–4^ Depending on the chemical composition, regional regulations, and geographic, geologic, and climatic conditions, these brines can be either discharged, disposed (via deep-well injection), or treated for minimal liquid discharge (MLD) or even zero liquid discharge (ZLD).^5,6^ ZLD refers to the approach of crystallizing out all of the salts for either valorization or solid waste disposal, regardless of whether the water is recovered.^7,8^ Brine management is often expensive, and the cost is typically dependent on the volume of brine to be discharged or subjected to ZLD treatment.^2,9^ Therefore, one effective approach to minimize the cost of brine management is to use energy-efficient and cost-effective processes to reduce the brine volume.^2,3^

Developing energy-efficient brine volume reduction processes requires maximizing the use of nonevaporative processes.^3^ Reverse osmosis (RO), the state-of-the-art process for brackish and seawater desalination,^10^ has two major challenges in brine volume reduction, including mineral scaling^11–13^ and the very high salinity of certain brines with their osmotic pressures exceeding the working pressure of conventional RO systems (∼80 bar).^14^ To address the second challenge, extensive research has been devoted to developing different variants of RO that can further concentrate brines with high osmotic pressures.

One strategy that enables pressure-driven membrane processes for brine volume reduction is to reduce the transmembrane osmotic pressure difference so that water can permeate through the membrane even when the applied hydraulic pressure is below the brine osmotic pressure. Among the multiple approaches for implementing such a strategy, low-salt-rejection RO (LSRRO) stands out due to its compatibility with existing spiral-wound membrane module design and the absence of internal concentration polarization that limits the performance of the alternative approach based on counter-flow RO.^15,16^ LSRRO uses conventional spiral-wound modules with “leaky”, low-salt-rejection (LSR) membranes that allow a certain degree of salt passage.^17^ The difference in osmotic pressures across the LSR membrane can be substantially lower than that across a typical RO membrane, which enables a concentration of high-salinity brines using moderate pressures (Figure 1). In a typical LSRRO system, brine from a high-rejection RO stage is further concentrated using sequential LSR modules with progressively lower salt rejection, while permeate is recycled upstream,^17–19^ enabling high brine concentrations at moderate pressures.^18^

**Figure 1:**
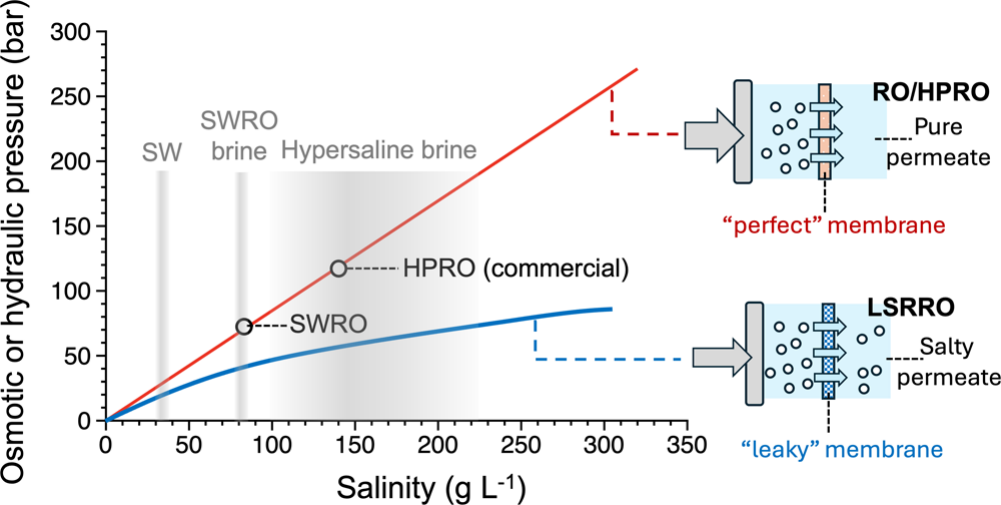
Illustration of the concept of LSRRO for concentrating a high-salinity brine. For RO membranes with nearly perfect salt rejection, the osmotic pressure is roughly linear with salinity (red line). To achieve a high level of salinity in the brine using an RO membrane, the applied hydraulic pressure needs to exceed the osmotic pressure of the brine, which is the case for RO or high-pressure RO (HPRO). LSRRO is an alternative strategy to achieve a high level of salinity by reducing the transmembrane osmotic pressure to enable water permeation even when the applied pressure is below the brine osmotic pressure (blue curve). Specifically, LSRRO uses a “leaky” membrane with relatively low salt rejection to reduce the transmembrane osmotic pressure, which enables treatment of high-salinity brines with a moderate applied pressure that is only a fraction of the brine osmotic pressure.

Early analyses of LSRRO relied on physically unrealistic assumptions such as constant salt rejection^17^ and a constant salt permeability coefficient (i.e., the *B* value),^18,20^ which are both known to vary with water flux and/or salinity. These limitations highlight the need for a physically grounded, closed-form model that contains only intrinsic parameters and remains valid across salinity ranges, where Donnan exclusion decreases. A model based on the solution-friction theoretical framework has recently been introduced by Du et al. to describe salt transport in LSR membranes.^21^ However, the key parameters in this formulation resembling the Spiegler–Kedem–Katchalsky (SKK) model, including the reflection coefficient, observed salt permeability, and friction factors, are not intrinsic properties. As shown in recent analyses of LSR membranes, these parameters vary strongly with interfacial feed salinity and require refitting at every bulk feed salinity and operating condition.^22^ This lack of intrinsic parametrization severely limits the predictive value of the SKK-like model for LSRRO and motivates the search for a closed-form analytical framework with true intrinsic membrane parameters.

Recently, Biesheuvel et al. proposed a novel analytical expression to describe the performance of brackish and seawater desalination with remarkable accuracy.^23,24^ The new analytical expression was also derived based on the solution-friction framework and is herein termed the solution-friction analytical approximation (SF-AA). The SF-AA features three intrinsic membrane parameters, including the water permeability coefficient, charge factor, and transport factor. The SF-AA represents a significant improvement over either the conventional framework with a constant *B* value or SKK-like formulations, because the three parameters in the SF-AA are intrinsic and can capture the dependence of membrane performance on salt concentration. The SF-AA has been experimentally validated up to seawater salinity (0.6 M). However, the SF-AA was developed and tested only for RO membranes with nearly perfect rejection, and its applicability for LSR membranes and in the salinity range relevant to LSRRO remains unknown.

The goal of this work is to adapt the SF-AA to LSR membranes and examine its applicability in describing LSRRO behavior in a salinity range much wider than that of conventional seawater or brackish water RO. We first present the SF-AA with modifications for LSR membranes. We then perform a series of experiments using three different membranes with different properties, including two commercial nanofiltration (NF) membranes and one chlorinated NF membrane, to measure the water flux and salt rejection under different solution and operating conditions. We fit the data set using the SF-AA to demonstrate its ability to describe the behavior of LSR membranes over a wide range of salinities (up to 3.6 M NaCl). Through this work, we aim to establish the SF-AA as a robust theoretical framework for LSRRO.

## Theory

The widely adopted framework for modeling RO processes uses the water permeability coefficient (*A*) and salt permeability coefficient (*B*) to relate water and salt fluxes to their respective driving forces:^25^

1 $$J_{w}=A\left(\Delta P-\Delta \pi_{m}\right)$$

2 $$J_{s}=B\Delta c_{m}$$

where Δ*P* is the applied hydraulic pressure, Δ*π*_m_ is the transmembrane osmotic pressure difference, and Δ*c*_m_ is the transmembrane salt concentration difference. The osmotic pressure difference was calculated accounting for solution nonideality (section S1 of the Supporting Information and Figure S1).^26^ Equations 1 and 2 have often been perceived as the main model framework based on the solution-diffusion theory,^25^ where the *A* and *B* values are often treated as constants, despite strong experimental evidence showing the strong dependence of *B* on feed salinity.^27^ Herein, we refer to the framework based on eqs 1 and 2 as the *A*/*B* framework.

The solution-friction (SF) model describes the steady-state transport of water and ions as a balance between frictional forces and driving forces established based on a chemical potential gradient.^27^ The ion flux in the SF model is governed by convection, diffusion, and electromigration mechanisms. For 1:1 salt, an analytical approximation of the salt flux in the SF model has been proposed, allowing for extraction of intrinsic membrane properties that are invariant with respect to feed salinity and water flux.^23^ Specifically, the salt flux in SF-AA can be expressed as follows

3 $$J_{s}=P\left(\sqrt{C^{2}+{c_{m}}^{2}}-\sqrt{C^{2}+{c_{p}}^{2}}\right)$$

where *P* is the transport factor (liters per square meter per hour), *C* is the charge factor (molar), *c*_m_ is the salt concentration at the membrane surface (molar), and *c*_p_ is the permeate concentration (molar). In its current form, eq 3 can be applied only for solutions containing a 1:1 electrolyte (i.e., not a multivalent electrolyte or mixed salts). Its derivation assumes that *P* and *C* are intrinsic membrane properties that are invariant with salinity, representing a clear advantage over SKK-like formulations that require concentration-dependent parameters.^22^ Consequently, the SF-AA framework enables prediction of salt transport across a wide salinity range without refitting parameters, whereas the SKK-like formulation is inherently local to specific operating conditions.

From eq 3, an analytical expression can be derived to relate the experimentally observed salt permeability coefficient, *B*_obs_ (liters per square meter per hour), to the feed concentration at the membrane surface and permeate concentration:

4 $$B_{\mathrm{obs}}=\frac{P\left(\sqrt{C^{2}+{c_{m}}^{2}}-\sqrt{C^{2}+{c_{p}}^{2}}\right)}{c_{m}-c_{p}}$$

We note that *P* is essentially the limiting *B*_obs_ value when the membrane is uncharged. In other words, the ion flux equation for the SF model (eq 3) can be reduced to that for the *A*/*B* framework (eq 2) if membrane charge plays a negligible role in ion transport. Concentration polarization (CP) was accounted for using the conventional film model (section S2).

## Materials and Methods

All chemicals were purchased from Sigma-Aldrich (St. Louis, MO). Sodium chloride (NaCl, 99.0%) was used to adjust the feed concentrations. A sodium hypochlorite solution (NaOCl, reagent grade, ≈12.5% available chlorine) was diluted to 15 000 ppm for chlorination of the polyamide membranes. The commercial NF membranes included NF90 (DuPont) and TS80 membranes (TriSep/Sterlitech). All membranes were immersed in deionized (DI) water for 24 h before use.

To show the wide range of applicability of the SF-AA, we tested three membranes with very different properties, including the tightest NF90 membrane, a slightly leakier TS80 membrane, and a very leaky membrane prepared by chlorination of the NF90 membrane (the c-NF90 membrane). To obtain the c-NF90 membrane, controlled chlorine treatment of NF90 membranes under an alkaline condition was performed to facilitate the hydrolysis of the polyamide layer, thereby increasing the salt permeability.^21,28^ Specifically, the NF90 membrane was placed in a cross-flow cell with the polyamide active layer facing the feed channel through which a NaOCl solution (15 000 ppm, pH 11) was recirculated by a peristaltic pump for 150 min. The membranes were then removed from the cell, rinsed repeatedly with DI water, and stored in a plastic container filled with DI water for subsequent tests. Following chlorination, physicochemical characterization was performed to verify the intended modification of the NF90 membrane. The electrical resistance, ζ potential, and ATR-FTIR measurements suggest that chlorination leads to increased ionic permeability, a more negative surface charge, and chemical modification of the polyamide active layer. Detailed characterization methods and data are provided in section S2 and Figure S2.

## Results and Discussion

Experimental data for all three tested membranes (NF90, TS80, and c-NF90) under various feed salinities and applied pressures are summarized in Figure 2, which shows that the simple SF-AA with three parameters provides satisfactory fitting for the experimental data (section S3 for the data-fitting procedure and section S4 for estimation of the mass transfer coefficient). Regardless of the membrane type and feed salinity, increasing the applied pressure increased the water flux (Figure 2a1–c1) and observed salt rejection (Figure 2a2–c2). Increasing the applied pressure increased both water flux and observed salt rejection, with rejection enhancement dominated by permeate dilution rather than increased salt flux.^27^

**Figure 2:**
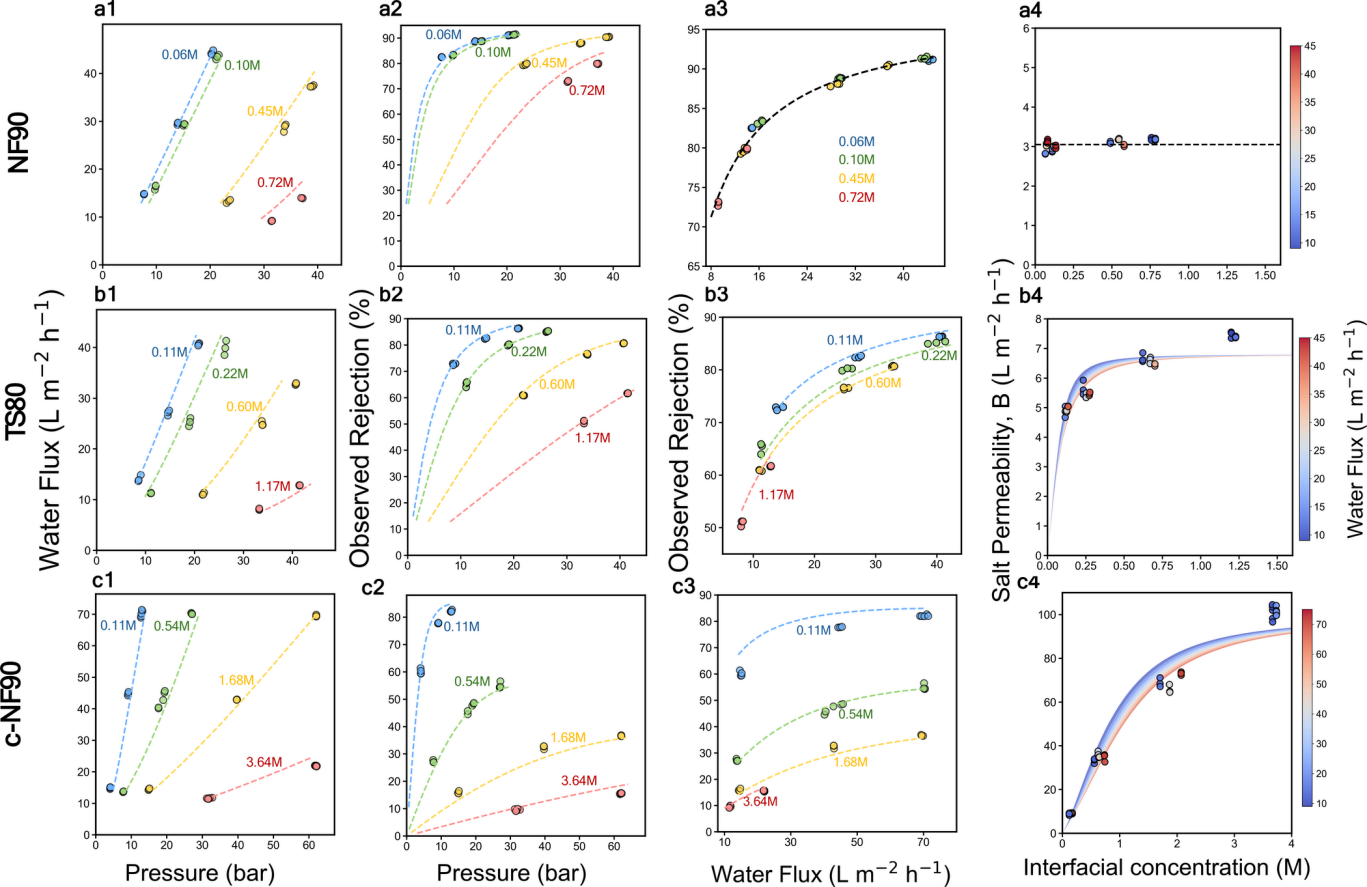
Fitting of experimental data using the SF-AA framework for (a1–c1) water flux as a function of applied pressure, (a2–c2) observed rejection as a function of applied pressure, (a3–c3) observed rejection as a function of water flux, and (a4–c4) observed salt permeability coefficient, *B*_obs_, as a function of interfacial concentration. The first (a1–a4), second (b1–b4), and third (c1–c4) rows of data represent results for NF90, TS80, and c-NF90 membranes, respectively. The experimental data were collected across a range of bulk feed concentrations and applied pressures, which differ by membrane, to yield a reasonable range of water flux. Filled circles represent experimental data collected, and dashed curves are the fitted results based on the SF-AA. Mass transfer coefficients of 140 L m^–2^ h^–1^ (NF90 and c-NF90) and 167 L m^–2^ h^–1^ (TS80) were used to account for concentration polarization. The fitting parameters for each membrane, including the water permeability coefficient (*A*), charge factor (*C*), and transport factor (*P*), are summarized in Figure 3a.

Plotting the observed rejection against water flux reveals very different behaviors of the three membranes (Figure 2a3–c3). Specifically, experiments with TS80 and c-NF90 membranes revealed distinct and feed salinity-dependent curves that relate observed rejections as a function of permeate fluxes (Figure 2b3,c3), but such curves appear to collapse into one master curve for the NF90 membrane (Figure 2a3). This observation suggests that feed salinity has strong impacts on the fundamental behavior of salt transport across the TS80 and c-NF90 membranes but not the NF90 membrane.

For the NF90 membranes, feed salinity affects salt rejection mainly through its impact on osmotic pressure and, consequently, permeate flux. This is evidenced by the constant observed salt permeability (*B*_obs_) regardless of the interfacial concentration (Figure 2a4). In contrast, the feed salinity has a strong impact on the salt transport behavior of the TS80 and c-NF90 membranes, as clearly indicated by the strong dependence of the observed salt permeability on interfacial concentration (Figure 2b4,c4). Such a dependence is particularly strong for the c-NF90 membrane whose *B*_obs_ increases from 9 L m^–2^ h^–1^ bar ^–1^ at 0.11 M to more than 100 L m^–2^ h^–1^ bar ^–1^ at 3.6 M.

Because *B*_obs_ is independent of salt concentration, the data set for the NF90 membrane can be fitted with an *A*/*B* framework that can be obtained by simplifying the SF-AA with a zero charge factor (*C*) in eq 4. However, for TS80 and c-NF90, the full SF-AA must be used. A charge factor of zero has also been reported for BW30 and XLE membranes in previous publications.^23^ Although all of these polyamide membranes (i.e., NF90, BW30, and XLE) are known to be negatively charged based on ζ potential measurements,^29^ the ζ potential reflects surface electrokinetic behavior and does not directly quantify the effective volumetric charge participating in ion exclusion within the membrane.^30,31^ According to a recent study, a substantial fraction of ionizable carboxyl groups within polyamide membranes remain un-ionized under typical operating conditions due to nanoconfinement and dielectric effects.^32^ As a result, electrostatic (Donnan) exclusion can be strongly suppressed even in intrinsically charged membranes, and salt transport becomes dominated by steric and solution-friction mechanisms.

The solution-friction model describes Donnan partitioning explicitly while lumping other contributions into a non-Donnan partitioning term, which provides an approximate analytical solution that is simple and elegant and can capture the dependence of the salt permeability coefficient on feed salinity (Figure 2b4,c4). The validity of the SF-AA framework for these LSR membranes is further evidenced by the collapse of experimental data onto a single master curve when plotting the observed salt permeability (*B*_obs_) against the interfacial concentration (*c*_m_). As suggested in derivations of the original SF-AA for RO membranes,^23^ the existence of such a master curve confirms that intrinsic parameters *P* and *C* are sufficient to describe the transport physics, even in regimes where the membrane is highly “leaky”. Within the SF-AA framework, the pronounced increase in *B*_obs_ for highly leaky membranes is consistent with reduced Donnan exclusion at high salinity, as eq 4 suggests that *B*_obs_ will asymptotically approach the limiting salt permeability coefficient, or the transport factor, *P*, as *c*_m_ increases. The water permeability coefficient (*A*), transport factor (*P*), and charge factor (*C*) for all three membranes are summarized in Figure 3a. As the feed salinity increased, the *B*_obs_ values for the TS80 and c-NF90 membranes approached their respective transport factors.

**Figure 3:**
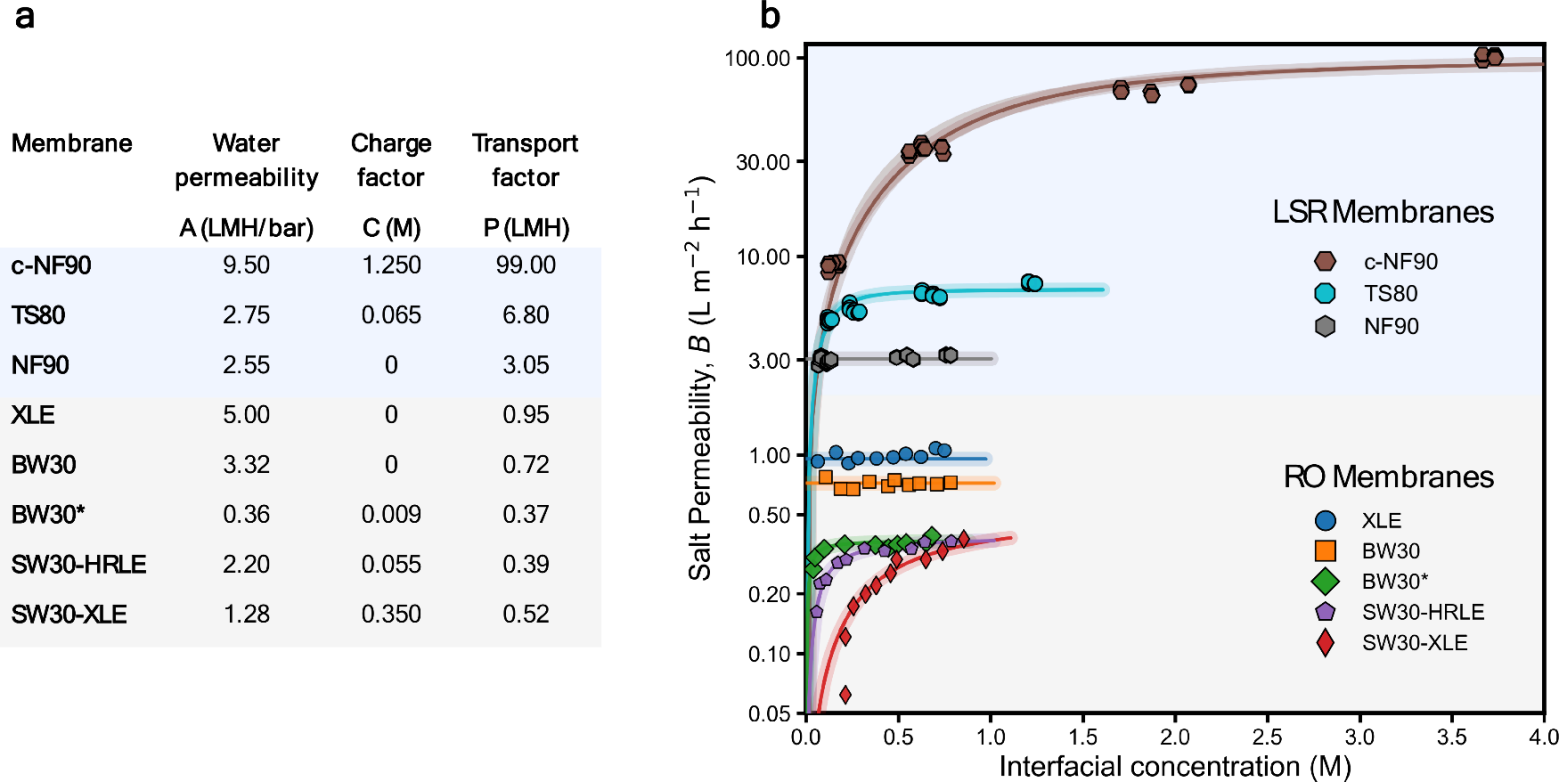
(a) Parameters of the SF-AA for the three LSR membranes tested in this work (NF90, TS80, and c-NF90) and other RO membranes reported in the literature. (b) Comparison of fitted and experimentally measured observed salt permeability coefficients (*B*_obs_) as a function of interfacial concentration for NF90, TS80, and c-NF90 membranes and other RO membranes reported in the literature. For the LSR membranes, data points are colored by water flux to illustrate flux-dependent behavior. The membrane marked with an asterisk was taken from ref 33.

With the original form of the SF-AA in which the permeate concentration (*c*_p_) was set to zero^23^ (i.e., with almost perfect salt rejection), *B*_obs_ is theoretically a function of only the interfacial salt concentration, *c*_m_ (section S5). In other words, the impact of water flux on *B*_obs_ was mainly exerted through its influence on *c*_m_. However, when the membrane is leaky and *c*_p_ is non-negligible, the relationship between *B*_obs_ and *c*_m_ becomes slightly dependent on the water flux. As water flux increases, *B*_obs_ decreases despite an increased salt flux, because the higher water flux enhances dilution on the permeate side, which in turn leads to a reduced *B*_obs_ according to eq 4. This additional influence of water flux on *B*_obs_ (beyond that through the impact of *c*_m_) is theoretically present but quantitatively small.

Comparing the *B*_obs_ values among the three membranes tested in this work as well as other RO membranes tested in the literature^23,33^ (Table S1 for testing conditions) suggests that the transport factors of all of the LSR membranes are substantially higher than those of any RO membranes for brackish water or seawater desalination (Figure 3b). Specifically, the lowest transport factor for the NF90 membrane (*P* ∼ 3 L m^–2^ h^–1^ bar ^–1^), the least leaky of all three membranes tested in this work, is 3 times higher than that of the most permeable RO membrane previously tested (XLE; *P* ∼ 1 L m^–2^ h^–1^ bar ^–1^) and almost an order of magnitude higher than that of seawater RO membranes (SW30; *P* ∼ 0.4 L m^–2^ h^–1^ bar ^–1^). The c-NF90 membrane yields a remarkably high transport factor of ∼99 L m^–2^ h^–1^ bar ^–1^, substantially exceeding that for TS80, NF90, and all RO membranes. While such a membrane would be too leaky for practical LSRRO implementation, c-NF90 serves as a critical stress test for the SF-AA framework. Notably, even at a very high salinity, where the selectivity of c-NF90 is severely compromised, the SF-AA model remains reasonably predictive. This result suggests that the SF-AA framework is robust across a wide range of membrane permeabilities and is, therefore, likely applicable to membranes with properties appropriate for LSRRO applications.

## Implications

We have demonstrated that the SF-AA is a simple yet highly robust model for describing the behavior of LSR membranes. The original form of the SF-AA has been shown to be effective in describing the concentration-dependent salt permeability coefficient for various RO membranes, using only one additional parameter compared to the conventional *A*/*B* framework. The current work extends the SF-AA to LSR membranes and demonstrates its applicability even for an extremely leaky membrane (c-NF90) up to a very high salinity (3.6 M NaCl). The SF-AA is powerful as a simple, closed-form analytical model that applies to a variety of salt-rejecting membranes over a broad salinity range. However, it should be recognized that the derivation of SF-AA considers only the impact of salinity on Donnan partitioning. Other possible mechanisms, such as salinity-dependent ion hydration behavior and polymer swelling, may also contribute to the observed behavior but are not explicitly distinguished in the present formulation. In addition, the present formulation was derived and validated for 1:1 salts, and its extension to multivalent salts or mixed electrolytes remains an open question that warrants future investigation.

While the specific c-NF90 membrane developed in this work may be too leaky even for LSRRO, the chlorination method used can likely generate LSR membranes with a wide range of properties via tuning the NaClO concentration and treatment time. The SF-AA is instrumental in characterizing any commercial or lab-modified membranes and generating a set of three parameters for each system (of membrane and salt) that can be used to predict water and salt fluxes at different feed concentrations and pressures. The simple yet robust SF-AA can also be applied for module-scale modeling to capture the spatial distributions of concentrations and fluxes along the module, providing a more accurate yet easily implementable model framework compared to those based on constant rejection or constant salt permeability coefficients. Specifically, because its parameters remain constant despite drastic changes in the feed concentration, the SF-AA framework is uniquely suited for modeling full-scale LSRRO modules. Unlike empirical models or SKK approaches that would require stepwise parameter adjustments along the module length to account for increasing salinity, the SF-AA allows for continuous, accurate simulation using a single set of parameters extracted from coupon-scale characterization.

## Supplementary Material



## Notes

Estimation of osmotic pressure based on the Pitzer model (section S1), film model for concentration polarization (section S2), conditions for filtration experiments (section S3), impact of chlorination on NF90 membrane properties (section S4), data fitting procedure (section S5), derivation of the observed salt permeability for high-rejection membranes (section S6), and summary of membrane test conditions (Table S1) (PDF)
