# Supplementary material for: Solution-Friction Analytical Approximation as a Robust Model Framework for Low-Salt-Rejection Reverse Osmosis
Source: Environ Sci Technol Lett. 2026 Jan 15;13(2):310–5. doi: 10.1021/acs.estlett.5c01130 (PMC12895524; doi:10.1021/acs.estlett.5c01130)
Supplement: Supplementary file 1 [file ez5c01130_si_001.pdf]

Supporting Information for

Solution-Friction Analytical Approximation as a Robust Model Framework for Low-Salt-Rejection Reverse Osmosis

Manuscript Submitted to

Environmental Science & Technology Letters

Rayan Alghanayem[a,b] , Weifan Liu[c], Rui Chen[c], and Shihong Lin* [a,c]

[a] Department of Chemical and Biomolecular Engineering, Vanderbilt University, Nashville, Tennessee 37235-1831, USA

[b] Department of Chemical Engineering, College of Engineering, King Saud University, P.O. Box 800, Riyadh11421, Saudi Arabia

[c] Department of Civil and Environmental Engineering, Vanderbilt University, Nashville, Tennessee 37235-1831, USA

* Email: shihong.lin@vanderbilt.edu

This Supporting Information file includes:

- osmotic pressure estimation based on the Pitzer model (Section S1)
- film model for concentration polarization (Section S2);
- conditions for filtration experiments (Section S3);
- impact of chlorination on NF90 membrane properties (Section S4);
- data fitting procedure (Section S5);
- derivation of the observed salt permeability for high-rejection membranes (Section S6);
- summary of membrane test conditions (Table S1).

S1. Osmotic pressure estimation based on the Pitzer model

The following equation represents the osmotic pressure (π) in bar as a function of NaCl concentration (c) in mol L^{-1} and is derived from the Pitzer model[1],

$$\pi = 5.08c^2 + 39.68c + 1.22 \quad \text{(S1)}$$

It is valid at a temperature of 21 °C for NaCl concentrations up to approximately 4 mol L^{-1}.

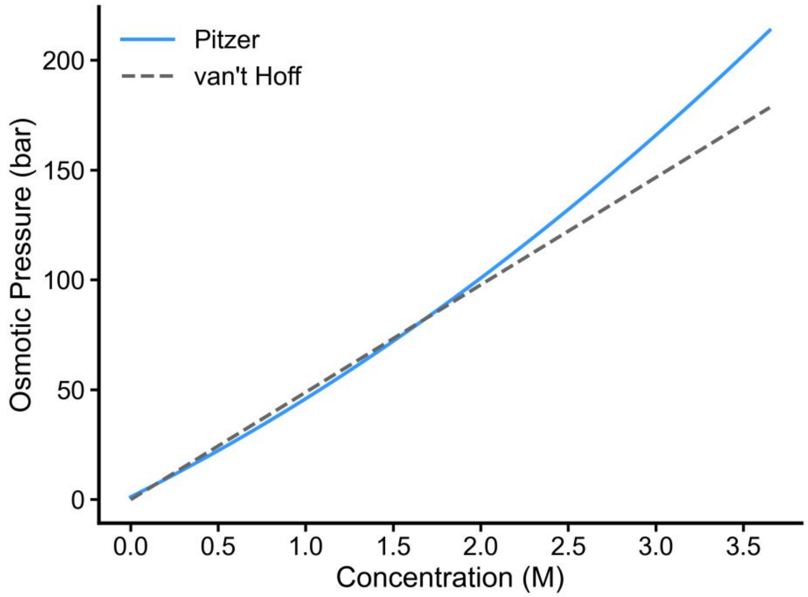

Figure S1. Comparison of osmotic pressure predictions using the Pitzer model (solid blue line) and the ideal van't Hoff equation (dashed gray line) for NaCl solutions at 21 °C. The deviation between the two models becomes significant at concentrations above ~2 M, highlighting the importance of accounting for non-ideality when modeling high-salinity brines in LSRRO processes.

S2. Film model for concentration polarization

Concentration polarization (CP) which is quantified via the CP modulus [2], f_{CP}:

$$f_{CP} \equiv \exp\left(\frac{J_w}{k}\right) = \frac{c_m - c_p}{c_f - c_p} \tag{S2}$$

where k is the mass transfer coefficient and depends on the hydrodynamic conditions, c_f and c_p are the bulk concentrations of the feed and permeate, respectively. Unlike typical desalination processes where c_p is negligible compared to c_f, c_p in LSRRO remains a significant fraction of c_f and thus the complete form of eq. 4 in the main text must be used instead of the simplified SF-AA experimentally validated in previous work for desalination membranes [3].

The feed-side mass-transfer coefficient k used in the CP calculations was estimated from a Sherwood-number correlation (Linton–Sherwood), consistent with the general approach of using Sherwood correlations to obtain k in RO/NF modeling[4].

$$Sh = \frac{k\, d_h}{D} = 0.023\, Re^{0.83} Sc^{1/3} \tag{S3}$$

where Sh is the Sherwood number, k is the mass transfer coefficient, d_h is the hydrodynamic diameter, D is the solute diffuision coefficient, ρ, and Re and Sc are the Reynold and Schmit numbers defined as:

$$Re = \frac{\rho\, u\, d_h}{\mu} \tag{S4}$$

$$Sc = \frac{\mu}{\rho D} \tag{S5}$$

where ρ is the fluid density, μ is the dynamic viscosity, and u is the crossflow velocity.

For a rectangular flow channel in a bench-scale cell for testing flat sheet membranes, the average crossflow velocity u was calculated from the volumetric flow rate Q, the channel width W, and channel height H:

$$u = \frac{Q}{WH} \tag{S6}$$

and the hydraulic diameter was evaluated as:

$$d_h = \frac{2WH}{W + H} \tag{S7}$$

S3. Conditions for filtration experiments

Bench-scale filtration experiments for NF90 and c-NF 90 membrane coupons were conducted using a high-pressure filtration system (Sterlitech, USA) with a CF042 membrane cell with active membrane area of 42 cm2 (9.2 cm × 4.6 cm) and crossflow velocity of 0.44 m s-1. A 31-mil Sterlitech diamond-pattern feed spacer was used in the CF042 feed channel. To demonstrate the applicability of the SF-AA framework across different testing configurations rather than to enable direct membrane-to-membrane performance comparison, the TS-80 membrane was tested in a small spiral-wound module with a crossflow rate of 6.6 L min^{-1}. The per-pass water recovery is negligible (<1%), maintaining essentially constant feed concentration and behaving like a coupon. NaCl solutions with varying concentrations were used as the feed solutions. The feed temperature was kept constant at 21 °C using a water bath (Thermo Fisher Scientific, USA).

For each of the three membranes (NF90, TS80, and c-NF90), we performed four sets of experiments with different feed salinities (from 0.06 to 3.64 M of NaCl, depending on membrane type). The feed salinity was adjusted by incrementally adding NaCl to the feed tank to reach different concentrations converted from measured conductivity. For a given membrane at a given feed salinity, we varied the applied pressure by adjusting the pressure-regulating valve to generate different permeate fluxes as monitored by a flowmeter (Tovatech). Samples of feed and permeate solution were collected and conductivity was measured with a portable conductivity meter (Fisher Scientific, USA) and converted to salt concentration using a pre-established calibration curve.

S4. Impact of chlorination on NF90 membrane properties

As shown in **Fig. S2a**, chlorination results in a substantial reduction in membrane electrical resistance. Measurements were conducted after removal of the polyester support layer using a four-electrode configuration, such that the measured resistance primarily reflects ion transport through the polyamide active layer. The observed decrease in resistance indicates enhanced ionic permeability of the chlorinated membrane relative to pristine NF90.

Zeta potential measurements (**Fig. S2b**) show that chlorination renders the membrane surface more negatively charged across the measured pH range. Compared to pristine NF90, the c-NF90 exhibits a systematic shift toward more negative zeta potential values, indicating a modification of surface charge characteristics following chlorine exposure.

ATR-FTIR spectra (**Fig. S2c**) further reveal chemical changes in the polyamide active layer after chlorination. Relative to pristine NF90, the c-NF90 membrane exhibits attenuation of characteristic amide I (~1660 cm^{-1}) and amide II (~1540 cm^{-1}) absorption bands, indicating modification of amide linkages in the polyamide network. Additional changes in the broad O–H/N–H stretching region (~3200–3600 cm^{-1}) are also observed, consistent with the formation of oxygen-containing functional groups following chlorination.

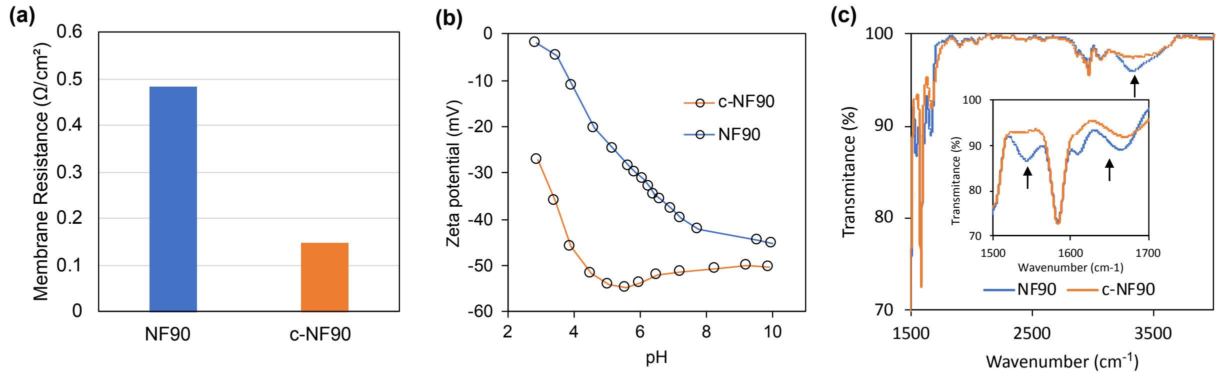

Figure S2. Physicochemical characterization of pristine and chlorinated c-NF90 membranes. **(a)** Electrical resistance of pristine NF90 and c-NF90 membranes measured after removal of the polyester support layer using a four-electrode configuration in 100 mM NaCl solution. Linear sweep voltammetry (LSV) was conducted with an applied voltage scan from −0.005 to 0.005 V. (b) Zeta potential of pristine NF90 and c-NF90 membranes measured as a function of solution pH in 1 mM KCl electrolyte. **(c)** Attenuated total reflectance Fourier transform infrared (ATR-FTIR) spectra of pristine NF90 and c-NF90 membranes, showing changes in characteristic polyamide absorption bands following chlorination.

S5. Data fitting procedure

We first determined the water permeability coefficient (A) for each membrane using filtration experiments with DI water, following the reduced form of eq. 1 ($J_w = A\Delta P$). With the A fixed for each membrane, we employed global optimization to extract two other intrinsic membrane properties(P and C) with k included as an additional fitted parameter for TS80; for NF90 and c-NF90, k was estimated from a Sherwood-number correlation. The optimization was performed subject to prescribed bounds for all membranes (P = 2–150 LMH; C = 0–2 M; k = 40–200 LMH). The global optimization was performed to search for one parameter set for each membrane such that the normalized sum of squared errors between simulations and measurements was minimized for water flux and salt flux from the same membrane (11 data points for each membrane). A weighted objective function was used to obtain stable and consistent fits across all membranes[5], recognizing that rejection and observed salt permeability are correlated observables and therefore do not provide independent constraints (i.e., the objective function combines errors in salt rejection and observed salt permeability). Different objective functions can also be formulated for this inverse modeling problem, but a detailed statistical treatment of optimal objective function selection is beyond the scope of this study.

S6. Derivation of the observed salt permeability for high-rejection membranes

We begin with a salt flux equation, based on the solution-friction model[6], that accounts for membrane charge and concentration polarization:

$$J_s = P\left(\sqrt{C^2 + c_m^2} - \sqrt{C^2 + c_p^2}\right) \tag{S8}$$

where J_s is salt flux, P is the transport factor, C is the charge factor, c_m is the salt concentration at the membrane interface, and c_p is the salt concentration on the permeate side[3]. The concentration at the membrane interface differs from the bulk feed concentration due to concentration polarization[2], described by the film model:

$$c_m = c_p + (c_f - c_p)\exp\left(\frac{J_w}{k}\right) \tag{S9}$$

where k is the mass transfer coefficient depends on the hydrodynamic conditions, c_f is the bulk concentrations of the feed, and J_w is water flux. For an RO membrane with near-perfect rejection, the permeate concentration c_p is very small and can be considered negligible. In this case, $\sqrt{C^2 + c_m^2} \sim C$, and Eq. S8 is reduced to:

$$J_s \approx P\left(\sqrt{C^2 + c_m^2} - C\right) \tag{S10}$$

The observed salt permeability is defined by:

$$B_{obs} = \frac{J_s}{(c_m - c_p)} \tag{S11}$$

For high-rejection membranes, since $c_p \ll c_m$ we can approximate $c_m - c_p \approx c_m$. Hence,

$$B_{obs} = \frac{P(\sqrt{C^2 + c_m^2} - C)}{c_m} \tag{S12}$$

Further simplification yields:

$$B_{obs} = P\left(\sqrt{\left(\frac{C}{c_m}\right)^2 + 1} - \frac{C}{c_m}\right) \tag{S13}$$

This expression[3] depends only on c_m and the intrinsic membrane parameters, membrane charge factor and membrane transport factor. Consequently, for high rejection membranes B_{obs} is governed primarily by c_m.

Table S1: Summary of membrane test conditions

Source dataset	Membrane	NaCl concentration range (M)	Temperature (°C)	Pressure (bar)
**This work**	NF90	0.06 – 0.72	21	10 – 40
	TS80	0.11 – 1.17		10 – 40
	c-NF90	0.11 – 3.64		4 – 60
**Ref. 3**	SW30-XLE	0.2 – 0.6	20 – 21	10 – 60
	SW30-HRLE	0.05 – 0.6		10 – 70
	BW30	0.1 – 0.6		5 – 60
	XLE	0.05 – 0.4		10 – 65
**Ref. 7**	BW30*	0.015 – 0.5	19	3 – 40

References:

1. Pitzer, K. S. Thermodynamics of electrolytes. I. Theoretical basis and general equations. *J Phys Chem* 77, 268–277 (1973).

2. Murthy, Z. V. P. & Gupta, S. K. *Estimation of Mass Transfer Coefficient Using a Combined Nonlinear Membrane Transport and Film Theory Model*. *Desalination* vol. 109 (1997).

3. Biesheuvel, P. M., Rutten, S. B., Ryzhkov, I. I., Porada, S. & Elimelech, M. Theory for salt transport in charged reverse osmosis membranes: Novel analytical equations for desalination performance and experimental validation. *Desalination* 557, 116580 (2023).

4. Treybal, R. Ewald. *Mass-Transfer Operations*. (McGraw-Hill, 1980).

5. Aster, R. C. ., Borchers, Brian. & Thurber, C. H. . *Parameter Estimation and Inverse Problems*. (Elsevier, 2019).

6. Wang, L. *et al.* Salt and Water Transport in Reverse Osmosis Membranes: Beyond the Solution-Diffusion Model. *Environ Sci Technol* 55, 16665–16675 (2021).

7. Blankert, B., Huisman, K. T., Martinez, F. D., Vrouwenvelder, J. S. & Picioreanu, C. Are commercial polyamide seawater and brackish water membranes effectively charged? *Journal of Membrane Science Letters* 2, (2022).
